# Sepsis of Patients Infected by SARS-CoV-2: Real-World Experience From the International HOPE-COVID-19-Registry and Validation of HOPE Sepsis Score

**DOI:** 10.3389/fmed.2021.728102

**Published:** 2021-10-14

**Authors:** Mohammad Abumayyaleh, Iván J. Nuñez-Gil, Ibrahim El-Battrawy, Vicente Estrada, Víctor Manuel Becerra-Muñoz, Aitor Uribarri, Inmaculada Fernández-Rozas, Gisela Feltes, Ramón Arroyo-Espliguero, Daniela Trabattoni, Javier López Pais, Martino Pepe, Rodolfo Romero, María Elizabeth Ortega-Armas, Matteo Bianco, Thamar Capel Astrua, Fabrizio D'Ascenzo, Oscar Fabregat-Andres, Andrea Ballester, Francisco Marín, Danilo Buonsenso, Raul Sanchez-Gimenez, Christel Weiß, Cristina Fernandez Perez, Antonio Fernández-Ortiz, Carlos Macaya, Ibrahim Akin

**Affiliations:** ^1^University Medical Centre Mannheim, University of Heidelberg, Mannheim, Germany; ^2^Hospital Clínico San Carlos, Universidad Complutense de Madrid, Instituto de Investigación, Sanitaria del Hospital Clínico San Carlos (IdISSC), Madrid, Spain; ^3^Hospital Clínico Universitario Virgen de la Vic, Málaga, Spain; ^4^Hospital Clínico Universitario de Valladolid, Valladolid, Spain; ^5^Hospital Severo Ochoa, Leganés, Spain; ^6^Hospital Nuestra Señora de América, Madrid, Spain; ^7^Hospital Universitario Guadalajara, Guadalajara, Spain; ^8^Centro Cardiologico Monzino, IRCCS, Milan, Italy; ^9^Complexo Hospitalario Universitario de Ourense, Spain; ^10^Azienda ospedaliero-universitaria consorziale policlinico di Bari, Bari, Italy; ^11^Hospital Universitario de Getafe, Universidad Europea, Madrid, Spain; ^12^Hospital General del norte de Guayaquil IESS Los Ceibos, Guayaquil, Ecuador; ^13^San Luigi Gonzaga University Hospital, Orbassano and Rivoli Infermi Hospital, Turin, Italy; ^14^Hospital Virgen del Mar, Madrid, Spain; ^15^San Giovanni Battista, Turin, Italy; ^16^Hospital IMED, Valencia, Spain; ^17^Hospital Clínico de Valencia, INCLIVA, Valencia, Spain; ^18^Hospital Clínico Universitario Virgen de la Arrixaca, IMIB-Arrixaca, CIBERCV, Murcia, Spain; ^19^Department of Woman and Child Health and Public Health, Fondazione Policlinico Universitario A. Gemelli IRCCS, Rome, Italy; ^20^University Hospital Joan XXIII, Tarragona, Spain; ^21^Department for Statistical Analysis, University Heidelberg, Mannheim, Germany; ^22^Complejo Hospitalario Universitario de Santiago de Compostela Instituto para la Mejora de la Asistencia Sanitaria (IMAS Fundación), Spain

**Keywords:** sepsis, score, COVID-19, SARS-CoV-2, outcome

## Abstract

**Background:** Patients with sepsis with a concomitant coronavirus (COVID-19) infection are related to a high morbidity and mortality rate. We investigated a large cohort of patients with sepsis with a concomitant COVID-19, and we developed a risk score for the estimation of sepsis risk in COVID-19.

**Methods:** We conducted a sub-analysis from the international Health Outcome Predictive Evaluation Registry for COVID-19 (HOPE-COVID-19-Registry, NCT04334291). Out of 5,837 patients with COVID-19, 624 patients were diagnosed with sepsis according to the Sepsis-3 International Consensus.

**Results:** In multivariable analysis, the following risk factors were identified as independent predictors for developing sepsis: current smoking, tachypnoea (>22 breath per minute), hemoptysis, peripheral oxygen saturation (SpO_2_) <92%, blood pressure (BP) (systolic BP <90 mmHg and diastolic BP <60 mmHg), Glasgow Coma Scale (GCS) <15, elevated procalcitonin (PCT), elevated troponin I (TnI), and elevated creatinine >1.5 mg/dl. By assigning odds ratio (OR) weighted points to these variables, the following three risk categories were defined to develop sepsis during admission: low-risk group (probability of sepsis 3.1–11.8%); intermediate-risk group (24.8–53.8%); and high-risk-group (58.3–100%). A score of 1 was assigned to current smoking, tachypnoea, decreased SpO_2_, decreased BP, decreased GCS, elevated PCT, TnI, and creatinine, whereas a score of 2 was assigned to hemoptysis.

**Conclusions:** The HOPE Sepsis Score including nine parameters is useful in identifying high-risk COVID-19 patients to develop sepsis. Sepsis in COVID-19 is associated with a high mortality rate.

## Introduction

The severe acute respiratory syndrome coronavirus 2 (SARS-CoV-2) outbreak, which was first emerged in Wuhan, China, in December 2019, has spread rapidly and has had an immense impact on the whole world. Consequently, states have endeavored to slow down the progression of the disease.

The course of coronavirus infectious disease (COVID-19) caused by SARS-CoV-2 is mild in the majority of patients. In 5% of COVID-19 patients, multiorgan dysfunction with an overall mortality rate of 1–11% was observed (1–4). However, sepsis is the main cause of death from the infection, particularly if not diagnosed and treated promptly.

It was revealed that many patients with severe COVID-19 showed general signs of shock (5). These patients met the sepsis and septic shock criteria according to the Sepsis-3 International Consensus (6). However, there are no comparative data available about the incidence and mortality rate in patients suffering from sepsis in COVID-19. In addition, predictors of sepsis have not yet been investigated.

In the international Health Outcome Predictive Evaluation Registry for COVID-19 (HOPE-COVID-19-Registry) (7), we compared baseline characteristics and clinical, laboratory, and radiologic findings in COVID-19 patients suffering from sepsis with those without sepsis at admission. We developed the HOPE Sepsis Score to estimate the risk of developing sepsis during admission. Predictors of mortality were analyzed.

## Methods

### Study Design and Patients

HOPE-COVID-19 (NCT04334291) is an international project. It is designed as a retrospective cohort registry without any financial compensation. The data of 5,837 consecutive hospitalized patients with COVID-19 were gathered. We analyzed all included patients from March 1, 2020, to June 2, 2020. An online database was built and completed by each participating center. Additional information on datasets of the HOPE-COVID-19-Registry is available at www.hopeprojectmd.com. The methodology of the HOPE-COVID-19-Registry has been described previously (7, 8). The study was approved by the Ethics Committee in all involved centers.

### Sepsis Definition III

The third international Consensus Task Force defined sepsis as life-threatening organ dysfunction due to a dysregulated host response to the infection. Organ failure in patients with sepsis increases in-hospital mortality by greater than 10% (6).

### Data Collection

Clinical laboratory investigation consisted of transaminases, glomerular filtration rate (GFR), creatinine, lactate dehydrogenase (LDH), electrolytes, coagulation profile, and complete blood count. Radiological imaging, such as chest radiography or CT, to detect bilateral or unilateral infiltrates was applied. Abnormal blood pressure (BP) was defined as systolic BP (SBP) less than 90 mmHg or diastolic BP (DBP) less than 60 mmHg. Glasgow Coma Scale (GCS) consisted of eye-opening, verbal, and motor responses. Elevated creatinine was defined as an elevation of more than 1.5 mg/dl, elevated troponin I (TnI) more than 0.05 μg/L, and procalcitonin (PCT) more than 0.5 ng/ml. We gathered as primary end point all-cause mortality. Oxygen therapy at admission including high nasal-cannula, non-invasive ventilation, and invasive mechanical ventilation, respiratory insufficiency, heart failure, upper respiratory tract involvement, clinically relevant bleeding, and embolic events as secondary end points were reported. Missing data are addressed in the tables.

### Statistical Analysis

Data of continuous variables were performed as mean ± SD with a normal distribution, median (interquartile range) with a non-normal distribution, while categorical variables were presented as frequencies and percentages (%). The Kolmogorov-Smirnov test was used to test the normal distribution. The Mann-Whitney U-test and Student's *t*-test were used to compare normal or non-normal distributions of continuous variables, respectively. For distribution analysis of categorical variables, Fisher's exact test or chi-squared test was used. We applied a two-tailed Fisher's exact test in tests with a sample size of *n* = 5 or below. Results are performed with 95% CIs. We estimated the differences in both groups using Kaplan-Meier and applied Log-Rank statistics. Predictors of sepsis were identified by univariate analysis. Predictors with *p* < 0.0001 were analyzed by the logistic multivariate regression. These variables were used to build a Score system. The Score system was confirmed through comparison with random choice with 10% of all the participants. Harrell's C-index or the area under the receiver operating characteristic curve (AUC-ROC) was used to evaluate the ability of risk scores to predict outcome (C-index measures the goodness of fit of a model, with 0.5 indicating no discrimination and 1.0 indicating perfect prediction). We estimated the mortality risk according to HOPE Sepsis Score using Kaplan-Meier and applied Log-Rank statistics. Sensitivity, specificity, and positive (PPV) and negative predictive values (NPV) of HOPE Sepsis Score to predict the sepsis in low-, intermediate-, and high-risk groups were calculated. Statistical analysis was showed with SPSS (IBM Statistics, Version 23.0. Armonk, NY: IBM Corp). *p* < 0.05 was recognized as statistically significant.

## Results

### Comparison of Sepsis to Non-Sepsis Participants

At baseline, patients suffering from sepsis in COVID-19 were older than non-sepsis patients (≥65 years old; 66.3 vs. 52%; *p* < 0.001). Patients with sepsis showed more baseline comorbidities, such as arterial hypertension (65.2 vs. 46.9%; *p* < 0.001), dyslipidemia (41.9 vs. 32.8%; *p* < 0.001), diabetes mellitus (DM) (25.6 vs. 17.7%; *p* < 0.001), and current smoking (11.4 vs. 4.5%; *p* < 0.001), Table 1. Clinical presentations, such as dyspnoea (68.1 vs. 55%; *p* < 0.001), tachypnoea (46.3 vs. 23.5%; *p* < 0.001), hemoptysis (6.3 vs. 1.1%; *p* < 0.001), anosmia or hyposmia (10.4 vs. 5.9%; *p* < 0.001), and dysgeusia (11.7 vs. 6.3%; *p* < 0.001), were more observed in the sepsis group as compared to the non-sepsis group. Clinical parameters at admission were worse in patients with sepsis as compared to non-sepsis patients with a decrease in peripheral oxygen saturation (SpO_2_) <92% and abnormal BP (systolic BP <90 mmHg and/or diastolic BP <60 mmHg; 61.1 vs. 31.1%; *p* < 0.001; and 16.8 vs. 5.8%; *p* < 0.001). Similarly, changes in laboratory parameters were also more pronounced in sepsis group (Table 1).

**Table 1:** Patients with Sepsis as compared to patients without Sepsis; Baseline characteristics, laboratory and radiographic findings, complications, and clinical outcomes.

**Characteristic**	**Patients with Sepsis *N* = 624**	**Patients without Sepsis *N* = 5213**	*P*-value*
Age – no. (%)			
<65	207/614 (33.7)	2458/5124 (47.9)	** <0.001**
≥65	407/614 (66.3)	2666/5124 (52)	** <0.001**
Male – no. (%)	417/624 (66.8)	3004/5213 (57.6)	** <0.001**
Duration of symptom onset to admission – days mean ± SD	5.9 ± 7.6	7.2 ± 6.5	** <0.001**
Duration of hospital stay – days mean ± SD	12.8 ± 11.7	9.8 ± 8.6	** <0.001**
Chronic conditions – no. (%)			
Arterial hypertension	407/624 (65.2)	2443/5213 (46.9)	** <0.001**
Dyslipidaemia	259/618 (41.9)	1643/5007 (32.8)	** <0.001**
Diabetes Mellitus	160/624 (25.6)	924/5213 (17.7)	** <0.001**
Obesity	126/497 (25.4)	890/4034 (22.1)	0.09
Current Smoking	71/624 (11.4)	235/5213 (4.5)	** <0.001**
Renal insufficiency ¥	75/624 (12)	306/5213 (5.9)	** <0.001**
Lung disease	126/624 (20.2)	933/5043 (18.5)	0.307
Cardiac disease	200/624 (32.1)	1129/5213 (21.7)	** <0.001**
Atrial Fibrillation	27/624 (4.3)	172/5043 (3.4)	0.24
Cerebrovascular disease	78/624 (12.5)	372/5213 (7.1)	** <0.001**
Connective Tissue disease	27/624 (4.3)	136/5213 (2.6)	**0.013**
Liver disease	30/624 (4.8)	182/5213 (3.5)	0.09
Cancer disease	131/624 (21)	639/5213 (12.3)	** <0.001**
Immunosuppression – no. (%) ≪	88/624 (14.1)	328/5213 (6.3)	** <0.001**
Prior tuberculosis – no. (%)	4/624 (0.6)	11/5043 (0.2)	0.074
Human Immunodeficiency virus – no. (%)	3/624 (0.5)	18/5043 (0.4)	0.498
Home Oxygen Therapy – no. (%)	31/624 (5)	140/5213 (2.7)	**0.002**
Premedication – no. (%)			
ASA Ω	148/624 (23.7)	720/5213 (13.8)	** <0.001**
Antiplatelet drug	36/579 (6.2)	166/4945 (3.4)	**0.001**
Oral Anticoagulation	98/624 (16.3)	494/5213 (9.5)	** <0.001**
Beta Blockers	147/601 (24.5)	761/4990 (15.3)	** <0.001**
Beta Agonist Inhalation Therapy	69/599 (11.5)	487/4983 (9.8)	0.178
Glucocorticoids Inhalation Therapy	58/604 (9.6)	438/4992 (8.8)	0.499
Vitamin D3	96/604 (15.9)	491/4966 (9.9)	** <0.001**
Benzodiazepine	115/606 (19)	729/4997 (14.5)	**0.004**
Antidepressant	104/603 (17.2)	625/4987 (12.5)	**0.001**
Symptomatic – no. (%)			
Asymptomatic	21/606 (3.5)	272/5012 (5.4)	**0.04**
Dyspnoea	425/624 (68.1)	2869/5213 (55)	** <0.001**
Tachypnoea > 22 breaths per minute	289/624 (46.3)	1226/5213 (23.5)	** <0.001**
Haemoptysis	39/624 (6.3)	57/5213 (1.1)	** <0.001**
Fatigue	315/589 (53.5)	2205/4896 (45)	** <0.001**
Anosmia / Hyposmia	65/624 (10.4)	310/5213 (5.9)	** <0.001**
Dysgeusia	73/624 (11.7)	329/5213 (6.3)	** <0.001**
Sorethroat	83/567 (14.6)	570/4779 (11.9)	0.062
Fever	511/614 (83.2)	3962/5003 (79.2)	**0.019**
Cough	398/606 (65.7)	3425/4992 (68.6)	0.143
Vomiting	49/586 (8.4)	358/4879 (7.3)	0.372
Diarrhea	105/581 (18.1)	965/4897 (19.7)	0.348
Erythromelalgia	157/579 (27.1)	1603/4880 (32.8)	**0.005**
Clinical parameters – no. (%)			
Peripheral Oxygen Saturation <92 %	375/624 (60.1)	1619/5213 (31.1)	** <0.001**
Reduced Blood Pressure §	105/624 (16.8)	302/5213 (5.8)	** <0.001**
GCS ø <15 – no. (%)	104/624 (16.7)	257/5213 (4.9)	** <0.001**
Laboratory parameters – no. (%) or median (IQR)			
Elevated Di-Dimer	410/624(65.7)	2773/5213(53.2)	** <0.001**
Elevated Procalcitonin	277/624 (44.4)	675/5213 (13)	** <0.001**
Elevated CRP ∂	581/624 (93.1)	4430/5213 (85)	** <0.001**
Elevated TnI ∞	126/624 (20.2)	279/5213 (5.4)	** <0.001**
Elevated Transaminases ∙	315/624 (50.5)	1836/5213 (35.2)	** <0.001**
Elevated Ferritin	272/390 (69.8)	1473/2552 (57.7)	** <0.001**
Elevated Triglyceride	100/345 (29)	416/2186 (19)	** <0.001**
Elevated LDH °	465/624 (82.2)	3247/5213 (62.1)	** <0.001**
Elevated Creatinine (>1.5 mg/dl)	211/624 (33.8)	596/5213 (11.4)	** <0.001**
Leukocytopenia (<4000 10E9/l)	79/619 (12.8)	739/4922 (15)	0.137
Lymphocytopenia (<1500 10E9/I)	469/605 (77.5)	3728/4832 (77.2)	0.839
Anemia hemoglobin (<12 g/dl)	232/624 (37.2)	1229/5213 (23.6)	** <0.001**
Thrombocytopenia (<150000 10E9/l)	193/611 (31.6)	1199/4908 (24.4)	** <0.001**
Moderate Hyponatremia	38/400 (9)	188/3876 (4.9)	** <0.001**
Severe Hyponatremia	19/624 (3)	39/5213 (0.7)	** <0.001**
Complication			
Respiratory Insufficiency	503/616 (81.7)	2302/5033 (45.7)	** <0.001**
Heart Failure	115/611 (18.8)	241/5012 (4.8)	** <0.001**
Acute kidney Injury	293/611 (48)	609/5026 (12.1)	** <0.001**
Upper Respiratory-Tract Infection	119/575 (20.7)	596/4959 (12)	** <0.001**
Pneumonia	575/624(92.1)	4471/5213(85.8)	** <0.001**
SIRS π	333/601 (55.4)	747/4991 (15)	** <0.001**
Any relevant bleeding ç	44/594 (7.4)	100/4978 (2)	** <0.001**
Embolic event	34/600 (5.7)	85/4999 (1.7)	** <0.001**
Oxygen Therapy			
O2 at the admission	563/621 (90.7)	3388/4958 (68.3)	** <0.001**
High Flow Nasal Cannula	283/604 (46.9)	798/4942 (16)	** <0.001**
Non-Invasive Mechanical Ventilation	206/603 (34.2)	545/4984 (11)	** <0.001**
Invasive Mechanical Ventilation	193/594 (32.5)	198/4955 (4)	** <0.001**
Another Medication or Intervention Procedures during the Admission			
Prone Position	156/599 (26)	400/4937 (8.1)	** <0.001**
ECMO å	4/396 (1)	21/3549 (0.6)	0.320
Use of Glucocorticoids	267/601 (44.4)	1243/4955 (25.1)	** <0.001**
Use of Hydroxychloroquine	483/608 (79.4)	4259/5013 (85)	** <0.001**
Use of Antiviral Drugs ∑	319/610 (52.3)	2978/4991 (59.7)	0.35
Use of Interferon	166/589 (28.2)	566/4940 (11.5)	** <0.001**
Use of Tocilizumab	126/592 (21.3)	330/4958 (6.7)	** <0.001**
Use of Antibiotics	530/593 (89.4)	3507/4727 (74.2)	** <0.001**
ACEI/ARB's ≪	110/587 (18.7)	963/4840 (19.9)	0.506
Anticoagulation	269/366 (73.5)	2182/2929 (74.5)	** <0.001**
Discharge			
ACEI/ARB's	69/624 (11.1)	924/5042 (18.3)	** <0.001**
Antiplatelet Drug	38/361 (10.5)	367/4336 (8.5)	0.180
Anticoagulation Drug	76/602 (12.6)	1018/4934 (20.6)	** <0.001**
Death †	382/624 (61.2)	767/5043 (15.2)	** <0.001**

***SD standard deviation**. ¥ CrCL <30. **≪** Immunosuppressive therapy for psoriasis arthritis, lung transplantation, kidney transplantation or systemic lupus erythematosus; oncological disease such as mamma-ca, prostate-ca, myelodysplastic syndrome or gammopathy; glucocorticoid therapy caused by COPD; dialysis; HIV or hepatitis. Ω Acetylsalicylic acid. **§** Systolic blood pressure <90 mmHg or diastolic blood pressure <60 mmHg. **Ø** Glasgow coma scale. **∂** C-reactive Protein. **∞** High sensitive Troponin I (cardiac injury; troponin > 99^th^ percentile upper reference limit). ∙ ALAT and ASAT. π Systemic inflammatory response syndrome. **Ç** Rectorrhagia, haematuria, epistaxis, and popliteal aneurysm bleeding with relevant decreased hemoglobin> 2 mg/l. **å** Extracorporeal membrane oxygenation. **∑** Lopinavir or /and Ritonavir. **≪** Premedication with ACEI/ARB's is not stopped. Significant p values are marked bold*.

### In-Hospital Course

Non-invasive ventilation and invasive mechanical ventilation were more often required in patients with sepsis as compared to those without sepsis, (34.2 vs. 11%; *p* < 0.001) and (32.5 vs. 4%; *p* < 0.001), respectively. Accordingly, the mortality rate was considerably higher in the sepsis group (61.2 vs. 15.2%; *p* < 0.001; Table 1).

### Treatment Approaches

During hospital stay, patients with sepsis more often received glucocorticoids (44.4 vs. 25.1%; *p* < 0.001), interferon (28.2 vs. 11.5%; *p* < 0.001), tocilizumab (21.3 vs. 6.7%; *p* < 0.001), and antibiotics (89.4 vs. 74.2%; *p* < 0.001). Interestingly, hydroxychloroquine use and antiviral drugs, such as lopinavir and/or ritonavir use, were higher in the non-sepsis group (79.4 vs. 85%; *p* < 0.001 and 52.3 vs. 59.7%; *p* = 0.35). Angiotensin-converting enzyme inhibitor (ACEi) or angiotensin receptor blocker (ARB) treatment at admission was not different in both groups (18.7 vs. 19.9%; *p* = 506; Table 1).

### Predictors of Sepsis, Development, and Validation of the HOPE Sepsis Score

**Figure 1:**
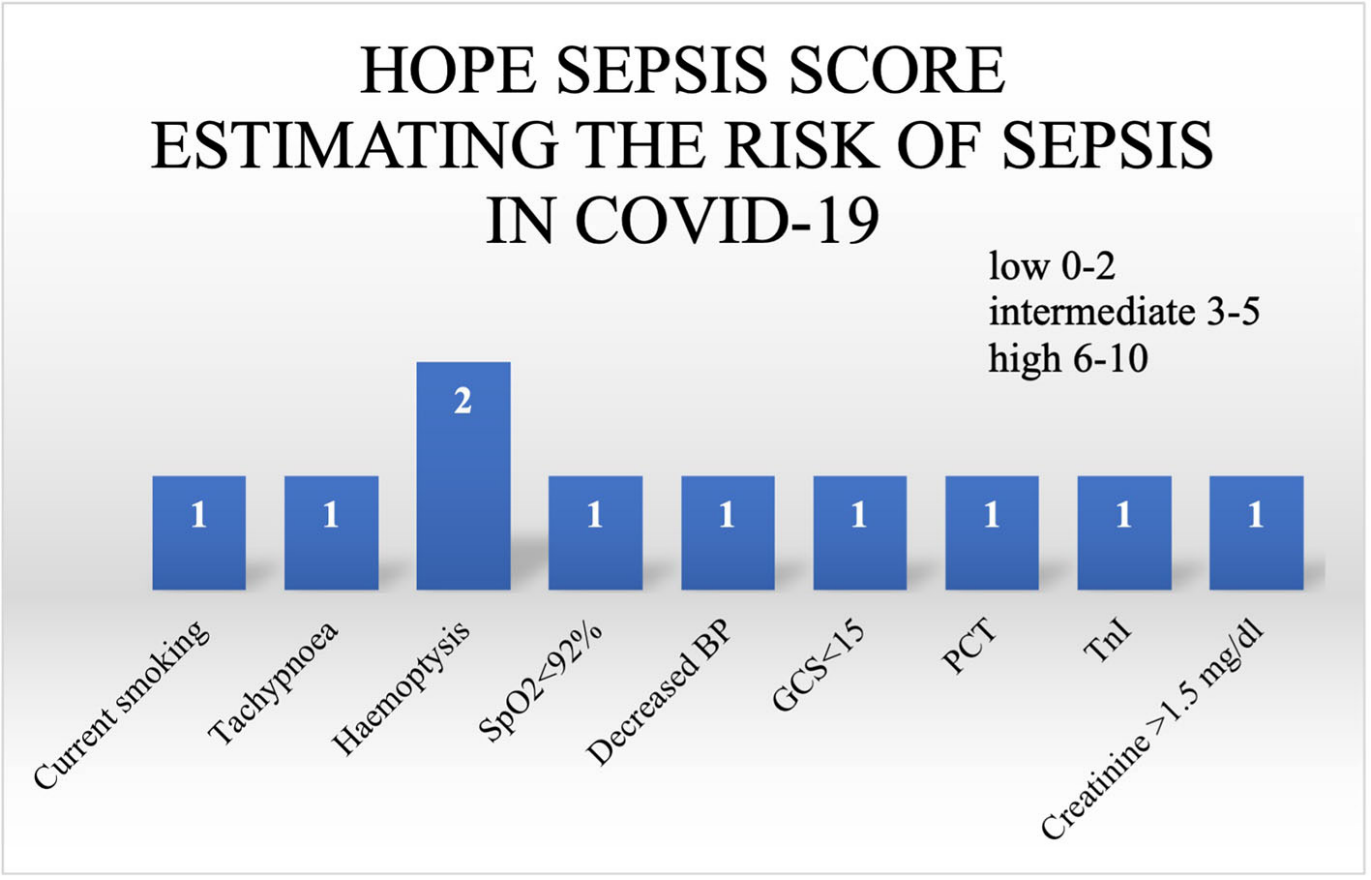
HOPE Sepsis Score, C-index = 0.763 (*N* = 5,837); tachypnoea >22 breath per minute; SpO_2_, peripheral oxygen saturation; BP, blood pressure; GCS <15 (Glasgow coma scale); PCT, elevated procalcitonin; TnI, elevated troponin; creatinine, elevated creatinine > 1.5 mg/dl; HOPE, the international Health Outcome Predictive Evaluation.

Table 2 presents the result of univariable and multivariable analyses. The multivariable analysis identified the following nine independent predictors to developing sepsis: current smoking (odds ratio, OR 2.43, 95% CI: 1.77–3.33; *p* < 0.001), tachypnoea (OR 1.60, 95% CI: 1.31–1.96; *p* < 0.001), hemoptysis (OR 4.30, 95% CI: 2.66–6.96; *p* < 0.001), reduced SpO_2_ <92% (OR 2.11, 95% CI: 1.73–2.57; *p* < 0.001), reduced BP at admission (OR 1.87, 95% CI: 1.08–3.22; *p* = 0.02), reduced GCS (OR 1.89, 95% CI: 1.42–2.51; *p* < 0.001), elevated PCT (OR 2.44, 95% CI: 1.99–2.99; *p* < 0.001), TnI (OR 1.94, 95% CI: 1.48–2.54; *p* < 0.001), and creatinine (OR 2.24, 95% CI: 1.81–2.78; *p* < 0.001). We divided the OR value of each variable by the median value of the regression coefficients of all variables (rounded to nearest 0.5 points). A score of 1 was assigned to current smoking, tachypnoea, decreased SpO_2_, decreased BP, decreased GCS, elevated PCT, TnI, and creatinine, whereas a score of 2 was assigned to hemoptysis. This score can be used to assess the risk for developing sepsis by assigning patients with COVID-19 to three risk groups: a low-risk group from 0 to 2 points, an intermediate-risk group from 3 to 5 points, and a high-risk group from 6 to 10 points (Figure 1). The probability of sepsis risk was 3.1–11.8% in the low-risk group, 24.8–53.8% in the intermediate-risk group, and 58.3–100% in the high-risk group.

**Table 2:** Predictors of Sepsis, multivariate analysis.

**Variable**	***Univariate analysis***			***Multivariate analysis***		
	**OR**	**95% CI**	***P*-value**	**OR**	**95% CI**	***P*-value**
Patients characteristic						
Male	1.48	1.24–1.76	** <0.001**			
Age≪	1.81	1.52–2.16	** <0.001**			
Chronic conditions						
Hypertension	2.13	1.79–2.53	** <0.001**			
Diabetes Mellitus	1.60	1.32–1.94	** <0.001**			
Current Smoking	2.72	2.06–3.59	** <0.001**	2.43	1.77–3.33	** <0.001**
Renal insufficiency	2.19	1.68–2.86	** <0.001**			
Prior heart disease	1.71	1.43–2.05	** <0.001**			
Cerebrovascular disease	1.86	1.43–2.41	** <0.001**			
Prior cancer disease	1.90	1.54–2.35	** <0.001**			
Connective tissue disease	1.69	1.11–2.57	**0.013**			
Previous therapies						
Immunosuppression	2.46	1.90–3.15	** <0.001**			
Home oxygen therapy	1.81	1.22–2.69	**0.002**			
ASA	1.94	1.59–2.37	** <0.001**			
Oral anticoagulation	1.78	1.41–2.25	** <0.001**			
Symptomatic						
Dyspnoea	1.75	1.46–2.08	** <0.001**			
Tachypnoea	2.81	2.37–3.32	** <0.001**	1.60	1.31–1.96	** <0.001**
Anosmia / Hyposmia	1.84	1.39–2.44	** <0.001**			
Dysgeusia	1.97	1.50–2.57	** <0.001**			
Haemoptysis	6.03	3.98–9.14	** <0.001**	4.30	2.66–6.96	** <0.001**
Clinical parameters at admission						
SpO2 <92%#	3.34	2.82–3.97	** <0.001**	2.11	1.73–2.57	** <0.001**
Reduced Blood Pressure §	3.29	2.59–4.18	** <0.001**	1.90	1.44–2.50	** <0.001**
Reduced GCS	3.86	3.02–4.93	** <0.001**	1.89	1.42–2.51	** <0.001**
Laboratory values						
Elevated CRP	2.38	1.74–3.29	** <0.001**			
Elevated Procalcitonin	3.84	3.20–4.61	** <0.001**	2.44	1.99–2.99	** <0.001**
Elevated Ferritin	1.88	1.59–2.24	** <0.001**			
Elevated LDH	1.77	1.47–2.14	** <0.001**			
Elevated Di-Dimer	1.69	1.42–2.01	** <0.001**			
Elevated TnI	4.47	3.56–5.63	** <0.001**	1.94	1.48–2.54	** <0.001**
Elevated Creatinine*	3.96	3.28–4.77	** <0.001**	2.24	1.81–2.78	** <0.001**
Elevated Transaminases	1.88	1.59–2.22	** <0.001**			
Anemia∑	1.91	1.61–2.28	** <0.001**			
Severe hyponatremia	4.17	2.39–7.26	** <0.001**			
X–Ray Abnormality						
Uni- or bilateral infiltrates	1.95	1.44–2.63	** <0.001**			

HR, hazard ratio; EF, ejection fraction; CI, confidence interval; ≪, age ≥ 65; *, > 1.5 mg/dl; ∂, <1500 10E9/I; ∑, Hb <12 g/dl; # peripheral oxygen saturation; **§** Systolic blood pressure <90 mmHg or diastolic blood pressure <60 mmHg. Significant p values are marked bold.

**Figure 2:**
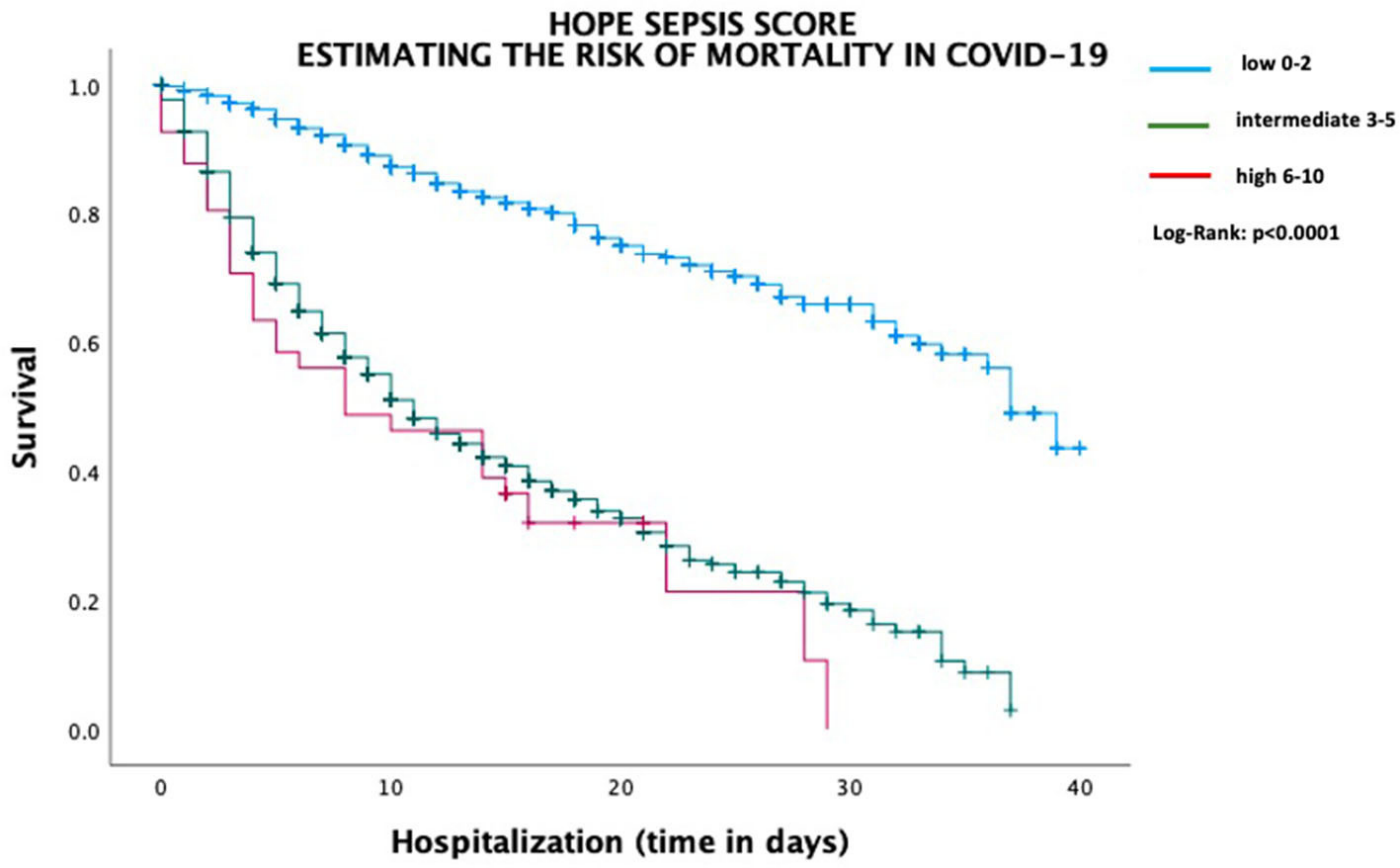
Estimating the risk of mortality in COVID-19 according to HOPE Sepsis Score.

The final model was applied to the validation cohort (random choice of 10% of all study participants). The C-index for the HOPE Sepsis Score was 0.763, while the C-index for the validation cohort was 0.77 (Table 3). In addition, the sensitivity of the HOPE Sepsis Score to predict sepsis was higher in the intermediate-risk group as compared to high-risk patients (81.1 vs. 34.3%). On the other hand, the specificity and PPV were lower in the intermediate-risk group than in patients with high risk for sepsis, respectively (specificity: 80.3 vs. 99.2% and PPV: 32.4 vs. 66.1%). In addition, estimating the risk of mortality in COVID-19 according to HOPE Sepsis Score was investigated (Figure 2). Clinical characteristics of the validated group, sensitivity, specificity, PPV, and NPV are presented in the Supplementary Materials.

**Table 3:** The validation of HOPE Sepsis Score; the Risk of developing sepsis in COVID-19 in the validated group (*n* = 584) as compared to all patients (*n* = 5837).

**Validated group**	**Findings at admission**	**Points**
***N* = 584**		
**Chronic conditions**	Current smoking	1
**Symptoms**	Tachypnoea	1
	Haemoptysis	2
**Clinical findings at admission**	SpO2 <92%	1
	Decreased BP	1
	GCS <15	1
**Laboratory values**	PCT	1
	TnI	1
	Creatinine >1.5 mg/dl	1

*C-index = 0.77 (N = 584); Tachypnoea >22 breath per minute; SpO2, peripheral oxygen saturation; BP, blood pressure; GCS, Glasgow coma scale; PCT, procalcitonin; TnI, troponin I*.

## Discussion

HOPE-COVID-19-Registry shows real-world experience from data worldwide. The present study shows patient characteristics at baseline, in-hospital complications, and mortality, particularly in the participants with sepsis. The main findings of the study are that (1) patients suffering from sepsis in COVID-19 had higher rates of comorbidity, (2) the incidence of sepsis in COVID-19 is estimated at 11%, (3) predictors for developing sepsis are identified, and (4) HOPE Sepsis Score is developed to support physicians to early identifying of COVID-19 patients with sepsis on the basis of chronic conditions, clinical findings, hemodynamic, and laboratory parameters at admission.

### Comparison of COVID-19 Patients With Sepsis and Without Sepsis

Patients with sepsis were older and had more comorbidities as compared to patients with non-sepsis. The incidence of sepsis in COVID-19 is estimated at 11%. In addition, in the sepsis cohort, an increase of inflammatory markers, such as CRP, PCT, and ferritin, was more pronounced than in participants with non-sepsis. This phenomenon is known in patients with sepsis due to excessive inflammation (9). In patients with COVID-19, the immune response seems to be more pronounced and may be based on underlying pathomechanisms: macrophage-activation syndrome, viral sepsis-induced immune paralysis, and dysregulation of an intermediate functional state of the immune system in infected patients with SARS-CoV-2 (10–12). Other laboratory abnormalities were more observed in participants with sepsis than those without sepsis, such as elevated d-dimer, transaminases, creatinine, LDH, anemia, thrombocytopenia, triglyceride, and hyponatremia. These abnormalities indicate that liver and kidney functions were impaired, such as coagulation disorder in patients with sepsis at admission. Clinical Data from 409 US hospitals from 2009 to 2014 in patients showed a slightly lower sepsis rate of 6% as compared to our data (13). Chen et al. reported that dead 119 patients with COVID-19 presented an increase of inflammatory parameters (14). The coagulation disorder may develop disseminated intravascular coagulopathy (DIC) in patients with sepsis. Therefore, it is proposed to establish prophylaxis against venous thromboembolism (VTE) (15). These changes, such as abnormal coagulation function, were observed in patients infected with SARS-CoV-2 (2, 14, 16). Additionally, COVID-19 patients have built antiphospholipid antibodies (17). However, the inflammation could increase procoagulant activity thereby contributing to thrombus formation (18). All these abnormalities may explain the higher rate of thromboembolism and multiorgan dysfunction in patients with sepsis.

## Hope Sepsis Score

HOPE Sepsis Score is developed and validated to support physicians to identify COVID-19 patients with sepsis. The score integrates nine parameters ranging from medical history to clinical and laboratory findings. Collecting the clinical findings, such as current smoking, hemoptysis, tachypnoea, decreased BP, GCS, SpO_2_, elevated PCT, TnI, and creatinine, at admission is relatively easy and promptly. Concerning this matter, a score of 2 is assigned to hemoptysis that represents an important predictor for developing sepsis. However, Hemoptysis is a less common symptom in patients with COVID-19 (1). As laboratory findings, the HOPE Sepsis Score represents TnI, PCT, and elevated creatinine as predictors for developing sepsis as compared to the sequential failure assessment (SOFA) score, which only included respiratory rate, GCS, BP, and elevated creatinine (6). To summarize, the HOPE Sepsis Score is also useful and feasible in identifying high-risk COVID-19 patients predicted to develop sepsis with a high mortality rate. The C-index for HOPE Sepsis Score was 0.763; the score can also be used to predict sepsis in COVID-19. The C-index of SOFA score in patients who required intensive care unit (ICU) was 0.74, while the C-index in other hospitalized patients was 0.79 (6). In addition, the C-index of qSOFA was 0.66 in ICU while it was 0.81 for non-ICU patients (19). The logistic organ dysfunction score (LODS) can be used to assessing the severity of sepsis in ICU. The C-index of LODS was 0.843 (20). In summary, the C-index of our score is comparable to the recently published scores. Additionally, the sensitivity of the HOPE Sepsis Score to predict sepsis was higher in intermediate as compared to high-risk patients (81.1 vs. 34.3%). On the other hand, the specificity and PPV of the HOPE Sepsis Score to predict the risk of sepsis were lower in patients with intermediate than those with high-risk for sepsis, respectively (specificity: 80.3 vs. 99.2% and PPV: 32.4 vs. 66.1%). However, the sensitivity and specificity of qSOFA ≥ 2 to predict in-hospital mortality were 69 and 55.5%, respectively (21). In 2,112 patients suffering from infections, the calculation of systemic inflammatory response syndrome (SIRS) and qSOFA showed a sensitivity of 52.8 and 19.5% and a specificity of 52.5 and 92.6% for 28-day mortality (22).

### Therapeutic Approaches in Patients With Sepsis in COVID-19

The use of antibiotic treatment was significantly higher in patients with sepsis than those without sepsis, followed by hydroxychloroquine and then antiviral drugs. Prone position was more revealed in sepsis as compared to patients with non-sepsis. The co-infection among COVID-19 patients with diverse co-pathogens including bacteria was reported (23). In one observational study, the treatment with hydroxychloroquine was not associated with a lower mortality rate (24). RECOVERY trial did not show a reduction of 28-day-mortality in patients with COVID-19 after lopinavir-ritonavir treatment (25). However, these patients did not suffer from sepsis. In addition, the short duration of prone position associated with better oxygenation did not improve the mortality rate (26). In other clinical trials, prone positioning for 16 hours every day in patients with acute respiratory distress syndrome (ARDS) was reduced to 90-day mortality (27). However, further randomized clinical trials are needed to investigate the safety and efficacy of all treatment options in patients infected by SARS-CoV-2.

### Outcomes of COVID-19 Patients With Sepsis

The mortality rate was significantly higher in patients with sepsis as compared to the non-sepsis group due to diverse complications (61.2 vs. 15.2%). In addition to respiratory insufficiency, other complications were more observed among patients with sepsis in comparison to non-sepsis participants; these included heart failure, acute kidney injury, pneumonia, bleeding, embolic event, and need for oxygen therapy including high flow nasal cannula, non-invasive, and invasive mechanical ventilation. In New York City, the mortality rate of COVID-19 patients, who received invasive mechanical ventilation, was less than the rate in our sepsis cohort (14.6%) but comparable with the non-sepsis group (28). Additionally, COVID-19 patients with cardiac injury presented a high mortality rate (51.2%) (29). In this regard, our data also showed that elevated TnI was associated with developing sepsis and consequently a high mortality rate. However, data in patients with sepsis with COVID-19 are limited.

At last, in comparison to SARS-CoV with 8,098 cases across 29 countries and Middle East respiratory syndrome (MERS) with 2,494 cases across 27 countries with the case-fatality rate (CFR) of 10% and 35%, the CFR of SARS-CoV-2 in Hubei was 2.9% and outside Hubei 0.4% with respect of challenges to identify all cases particularly with asymptomatic and mild courses (4, 30).

Therefore, patients with more comorbidities are susceptible to suffer from sepsis. Smokers who particularly suffering from hemoptysis and tachypnoea with decreased BP, SpO2, and GCS at admission who show abnormal laboratory as elevated PCT, TnI, and creatinine are more potential to develop sepsis when infected by SARS-CoV-2.

This study has some limitations. It has a retrospective character, not all laboratory tests were done in all patients. In addition, data about blood, urine, and stool culture are missing. External validation of our sepsis score is not performed.

## Data Availability Statement

The raw data supporting the conclusions of this article will be made available by the authors, without undue reservation.

## Ethics Statement

The studies involving human participants were reviewed and approved by University Medical Centre Mannheim, University of Heidelberg, Mannheim, Germany. Written informed consent for participation was not required for this study in accordance with the national legislation and the institutional requirements.

## Author Contributions

MA, IN-G, IE-B, and IA made substantial contributions to the study concept and design. All authors took obtaining ethical approval. Data were collected by MA, IN-G, IE-B, VE, VB-M, AU, IF-R, GF, RA-E, DT, JL, MP, RR, MO-A, MB, TA, FD'A, OF-A, AB, FM, DB, RS-G, CF, AF-O, and CM. MA, IN-G, and IA analysed all data. CW supported the descriptive statistics. IN-G approved the statistical analysis. MA, IN-G, IE-B, and IA prepared the manuscript. All authors contributed to the article and approved the submission version.

## Funding

Non-conditioned grant (Fundación Interhospitalaria para la Investigación Cardiovascular, FIC, Madrid, Spain). This nonprofit institution had no role in the study design; in the collection, analysis, and interpretation of data; in the writing of the report; nor in the decision to submit the paper for publication.

## Conflict of Interest

The authors declare that the research was conducted in the absence of any commercial or financial relationships that could be construed as a potential conflict of interest.

## Publisher's Note

All claims expressed in this article are solely those of the authors and do not necessarily represent those of their affiliated organizations, or those of the publisher, the editors and the reviewers. Any product that may be evaluated in this article, or claim that may be made by its manufacturer, is not guaranteed or endorsed by the publisher.
