# Supplementary material for: Sepsis of Patients Infected by SARS-CoV-2: Real-World Experience From the International HOPE-COVID-19-Registry and Validation of HOPE Sepsis Score
Source: Front Med (Lausanne). 2021 Oct 14;8:728102. doi: 10.3389/fmed.2021.728102 (PMC8603931; doi:10.3389/fmed.2021.728102)
Supplement: Supplementary file 1 [file Data_Sheet_1.docx]

Supplementary Appendix

| Table 1. Characteristics of all Patients at Baseline. | |
| --- | --- |
| Characteristic | All Patients n=5837 |
| Age –– yr no. (%) |  |
| < 65 | 2665/5737 (46.5) |
| ≥ 65 | 3073/5737 (53.6) |
| Duration of symptom onset to admission –– days mean ± SD | 7±6.6 |
| Duration of hospital stay –– days mean ± SD | 10±9 |
| Pregnancy ––no. (%) | 25/5837 (0.4) |
| Chronic conditions –– no. (%) |  |
| Lung disease | 1096/5837 (18.8) |
| Asthma | 308 (5.3) |
| COPD | 421 (7.2) |
| Interstitial | 38 (0.7) |
| Restrictive | 50 (0.9) |
| Others | 279 (4.8) |
| Cardiac disease | 1329/5837 (22.8) |
| Arrhythmias | 400 (6.9) |
| Coronary | 405 (6.9) |
| Cardiomyopathy | 121 (2.2) |
| Valvular | 130 (2.2) |
| Combined | 242 (4.1) |
| Others | 31 (0.6) |
| Cancer | 770/5722 (13.5) |
| Haematological | 97 (1.7) |
| Mamma | 73 (1.3) |
| Genitourinary | 241 (4.1) |
| Intestine | 128 (2.2) |
| Lung | 58 (1) |
| Pharyngolaryngeal | 10 (0.2) |
| Skin | 55 (1) |
| Other | 121 (2.1) |
| Premedication –– no. (%) |  |
| Beta Agonist Inhalation Therapy | 575/5719 (10.1) |
| Glucocorticoids Inhalation Therapy | 512/5737 (8.9) |
| Vitamin D3 | 598/5707 (10.5) |
| Benzodiazepine | 864/5743 (15) |
| Antidepressant | 746/5730 (13) |
| Severity of Dyspnoea –– no. (%) |  |
| None | 2429/5721 (43) |
| Mild | 1614/5721 (27.7) |
| Moderate | 1149/5721 (19.7) |
| Severe | 531/5721 (9.1) |
| Laboratory parameters –– no. (%) or median (IQR) |  |
| Elevated Di-Dimer | 3183/5837 (54.5) |
| Elevated Procalcitonin | 902/5837 (15.5) |
| Elevated CRP ∂ | 5011/5837 (85.9) |
| Elevated TnI ∞ | 405/5837 (7) |
| Elevated Transaminases • | 2151/5837 (36.9) |
| Elevated Ferritin | 1786/5837 (30.6) |
| Elevated Triglyceride | 522/2570 (20.3) |
| Elevated LDH º | 3712/5837 (63.6) |
| Elevated Creatinine >1.5 mg/dl | 807/5837 (13.8) |
| Natrium (mmol/l) –– median (min-max) | 138 (100-180) |
| Leukocytes (10E9/l) –– median (min-max) | 6240 (400-41100) |
| Lymphocytes (10E9/l) –– median (min-max) | 1000 (86-40000) |
| Haemoglobin (g/dl) –– median (min-max) | 14 (4-19) |
| Platelet (10E9/l) –– median (min-max) | 196000 (5000-950000) |
| X-Ray Abnormality –– no. (%) |  |
| Bilateral infiltrates | 3501/5667 (61.8) |
| Unilateral infiltrates | 1018/5667 (18) |
| No Abnormality | 1148/5667 (20.3) |
| Complication at admission –– no. (%) |  |
| Respiratory Insufficiency | 2867/5742 (49.9) |
| Heart Failure | 367/5694 (6.4) |
| Acute kidney Injury | 924/5712 (16.2) |
| Upper Respiratory-Tract Infection | 725/5588 (13) |
| Pneumonia | 5046/5837 (86.4) |
| SIRS π | 1103/5644 (19.5) |
| Sepsis | 624/5667 (11) |
| Any relevant bleeding ¥ | 145/5617 (2.6) |
| Embolic event | 120/5658 (2.1) |
| Oxygen Therapy at the admission –– no. (%) |  |
| O2 at the admission | 4053/5701 (71.1) |
| High Flow Nasal Cannula | 1119/5648 (19.8) |
| Non-Invasive Mechanical Ventilation | 780/5688 (13.7) |
| Invasive Mechanical Ventilation | 412/5645 (7.3) |
| Duration of Mechanical Ventilation in days –– mean ± SD | 8±6.6 |
| Another Medication or Intervention Procedures during the Admission –– no. (%) |  |
| Prone Position | 571/5625 (10.2) |
| ECMO å | 253/5632 (4.5) |
| Use of Glucocorticoids | 1547/5654 (27.4) |
| Use of Hydroxychloroquine | 4833/5730 (84.3) |
| Use of Antiviral Drugs ∑ | 3447/5714 (60.3) |
| Use of Interferon | 749/5626 (13.3) |
| Use of Tocilizumab | 472/5643 (8.4) |
| Use of Antibiotics | 4120/5446 (75.7) |
| ACEI/ARB’s « | 1090/5511 (19.8) |
| Anticoagulation Drug | 2481/3333 (74.4) |
| No | 852 (25.6) |
| Prophylaxis | 1874 (56.2) |
| Parenteral | 499 (15) |
| Anti-Vitamin K Antagonist | 69 (2.1) |
| Direct Oral Anticoagulation | 39 (1.2) |
| Discharge –– no. (%) |  |
| ACEI/ARB’s | 1002/5836 (17.2) |
| Antiplatelet Drug | 408/4761 (8.6) |
| Anticoagulation Drug | 1103/5699 (19.4) |
| Death † –– no. (%) | 1188/5837 (20.4) |

**SD standard deviation. ∂** C-reactive Protein. **∞** High sensitive Troponin I. • ALAT and ASAT. SARS-CoV-2; Severe acute respiratory syndrome. COVID-19; Coronavirus infectious disease 19. **º** Lactate dehydrogenase. π Systemic inflammatory response syndrome. **¥** Rectorrhagia, haematuria, epistaxis, and popliteal aneurysm bleeding with relevant decreased hemoglobin> 2 mg/l. **å** Extracorporeal membrane oxygenation. **∑** Lopinavir or /and Ritonavir. **«** Premedication with ACEI/ARB’s during hospital admission was not stopped.

| Table 2. Characteristics of the validated group at Baseline. | |
| --- | --- |
| Characteristic | Validated group n=584 |
| Age –– yr no. (%) |  |
| < 65 | 246/573 (42.9) |
| ≥ 65 | 327/573 (57.1) |
| Duration of symptom onset to admission –– days mean ± SD | 6.6±6.6 |
| Duration of hospital stay –– days mean ± SD | 13.9±11 |
| Pregnancy ––no. (%) | 0 (0) |
| Chronic conditions –– no. (%) |  |
| Arterial hypertension | 332/582 (57) |
| Dyslipidaemia | 217/580 (37.4) |
| Diabetes Mellitus | 122/584 (20.9) |
| Obesity | 123/491 (25.1) |
| Current Smoking | 46/544 (8.5) |
| Renal insufficiency ¥ | 45/584 (7.7) |
| Atrial Fibrillation | 26/584 (4.5) |
| Cerebrovascular disease | 45/573 (7.9) |
| Connective Tissue disease | 13/575 (2.3) |
| Liver disease | 26/576 (4.5) |
| Immunosuppression –– no. (%) « | 68/542 (12.5) |
| Prior tuberculosis –– no. (%) | 2/584 (0.3) |
| Human Immunodeficiency virus –– no. (%) | 4/584 (0.7) |
| Home Oxygen Therapy –– no. (%) | 19/580 (3.3) |
| Lung disease | 123/584 (21.1) |
| Asthma | 21 (3.6) |
| COPD | 54 (9.2) |
| Interstitial | 4 (0.7) |
| Restrictive | 6 (1) |
| Others | 38 (6.5) |
| Cardiac disease –– no. (%) | 157/584 (26.9) |
| Arrhythmias | 35 (6) |
| Coronary | 56 (9.6) |
| Cardiomyopathy | 14 (2.4) |
| Valvular | 14 (2.4) |
| Combined | 34 (5.8) |
| Others | 4 (0.7) |
| Cancer –– no. (%) | 83/578 (14.4) |
| Haematological | 11 (1.9) |
| Mamma | 4 (0.7) |
| Genitourinary | 29 (5) |
| Intestine | 11 (1.9) |
| Lung | 8 (1.4) |
| Pharyngolaryngeal | 3 (0.5) |
| Skin | 4 (0.7) |
| Other | 12 (2.1) |
| Premedication –– no. (%) |  |
| ASA Ω | 112/576 (19.4) |
| Antiplatelet drug | 28/567 (4.9) |
| Oral Anticoagulation | 66/571 (11.6) |
| Beta-Blockers | 121/574 (21.1) |
| Beta Agonist Inhalation Therapy | 52/574 (9.1) |
| Glucocorticoids Inhalation Therapy | 51/576 (8.9) |
| Vitamin D3 | 49/575 (8.5) |
| Benzodiazepine | 74/576 (12.8) |
| Antidepressant | 64/575 (11.1) |
| Severity of Dyspnoea –– no. (%) |  |
| None | 155/584 (18.5) |
| Mild | 168/584 (28.7) |
| Moderate | 153/584 (26.2) |
| Severe | 108/584 (18.5) |
| Laboratory parameters –– no. (%) or median (IQR) |  |
| Elevated Di-Dimer | 403/584 (69) |
| Elevated Procalcitonin | 138/584 (23.6) |
| Elevated CRP ∂ | 545/584 (93.3) |
| Elevated TnI ∞ | 82/584 (14) |
| Elevated Transaminases • | 283/584 (51.8) |
| Elevated Ferritin | 244/584 (41.8) |
| Elevated Triglyceride | 63/262 (44.9) |
| Elevated LDH º | 432/584 (74) |
| Elevated Creatinine >1.5 mg/dl | 126/584 (21.6) |
| Natrium (mmol/l) –– median (min-max) | 137 (111-167) |
| Leukocytes (10E9/l) –– median (min-max) | 7005 (648-28000) |
| Lymphocytes (10E9/l) –– median (min-max) | 900 (100-19100) |
| Haemoglobin (g/dl) –– median (min-max) | 14 (6-19) |
| Platelet (10E9/l) –– median (min-max) | 194000 (13800-752000) |
| X-Ray Abnormality –– no. (%) |  |
| Bilateral infiltrates | 488/584 (83.6) |
| Unilateral infiltrates | 68/584 (11.6) |
| No Abnormality | 28/584 (4.8) |
| Complication at admission –– no. (%) |  |
| Respiratory Insufficiency | 463/582 (79.6) |
| Heart Failure | 70/579 (12.1) |
| Acute kidney Injury | 168/582 (28.9) |
| Upper Respiratory-Tract Infection | 90/566 (15.9) |
| Pneumonia | 557/583 (95.5) |
| SIRS π | 251/575 (43.7) |
| Sepsis | 220/584 (37.7) |
| Any relevant bleeding ¥ | 43/576 (7.5) |
| Embolic event | 34/577 (5.9) |
| Oxygen Therapy at the admission –– no. (%) |  |
| O2 at the admission | 519/566 (91.7) |
| High Flow Nasal Cannula | 209/571 (36.6) |
| Non-Invasive Mechanical Ventilation | 231/574 (40.2) |
| Invasive Mechanical Ventilation | 161/577 (27.9) |
| Duration of Mechanical Ventilation in days –– mean ± SD | 5.5±7.2 |
| Another Medication or Intervention Procedures during the Admission –– no. (%) |  |
| Prone Position | 258/577 (44.7) |
| ECMO å | 100/574 (17.4) |
| Use of Glucocorticoids | 288/578 (49.8) |
| Use of Hydroxychloroquine | 504/579 (87) |
| Use of Antiviral Drugs ∑ | 403/578 (69.7) |
| Use of Interferon | 140/568 (24.6) |
| Use of Tocilizumab | 138/573 (24.1) |
| Use of Antibiotics | 480/550 (87.3) |
| ACEI/ARB’s « | 119/567 (21) |
| Anticoagulation Drug | 339/407 (83,3) |
| No | 68/407 (16.7) |
| Prophylaxis | 220/407 (54.1) |
| Parenteral | 108/407 (26.5) |
| Anti-Vitamin K Antagonist | 10/407 (2.5) |
| Direct Oral Anticoagulation | 1/407 (0.2) |
| Discharge –– no. (%) |  |
| ACEI/ARB’s | 88/584 (15.1) |
| Antiplatelet Drug | 36/378 (9.5) |
| Anticoagulation Drug | 111/575 (19.3) |
| Death † –– no. (%) | 260/584 (44.5) |

**SD standard deviation. ¥** CrCL < 30. **«** Immunosuppressive therapy for psoriasis arthritis, lung transplantation, kidney transplantation or systemic lupus erythematosus; oncological disease such as mamma-ca, prostate-ca, myelodysplastic syndrome or gammopathy; glucocorticoid therapy caused by COPD; dialysis; HIV or hepatitis. Ω Acetylsalicylic acid. **∂** C-reactive Protein. **∞** High sensitive Troponin I. • ALAT and ASAT. SARS-CoV-2; severe acute respiratory syndrome. COVID-19; Coronavirus infectious disease 19. **º** Lactate dehydrogenase. π Systemic inflammatory response syndrome. **¥** Rectorrhagia, haematuria, epistaxis, and popliteal aneurysm bleeding with relevant decreased hemoglobin> 2 mg/l. **å** Extracorporeal membrane oxygenation. **∑** Lopinavir or /and Ritonavir. **«** Premedication with ACEI/ARB’s during hospital admission was not stopped.

**Table 3. Sensitivity, specificity, positive, and negative predictive value of HOPE Sepsis Score to predict sepsis in low-, intermediate-, and high-risk groups.**

| Score | probability of sepsis | Sensitivity^1^ | Specificity^2^ | PPV^3^ | NPV^4^ |
| --- | --- | --- | --- | --- | --- |
| Low-risk group 0-2 | 3.1-11.8% | 78.2% | 47.9% | 9.5% | 96.9% |
| Intermediate-risk group 3-5 | 24.8-53.8% | 81.1% | 80.3% | 32.4% | 96.9% |
| High-risk group 6-10 | 58.3-100% | 34.3% | 99.2% | 66.1% | 96.9% |

^1^ Sensitivity of the score is defined as the ability to designate an individual suffering from disease as positive.^2^ Specificity of the score is defined as the ability to designate an individual not suffering from disease as negative. ^3^ PPV; Positive predictive value. ^4^ NPV; Negative predictive value.

**STROBE Statement**

|  | | Item No | Recommendation | Page No |
| --- | --- | --- | --- | --- |
| **Title and abstract** | | 1 | (*a*) Indicate the study’s design with a commonly used term in the title or the abstract | 1-3 |
|  |  |  | (*b*) Provide in the abstract an informative and balanced summary of what was done and what was found | 3-4 |
| Introduction | | | | |
| Background/rationale | | 2 | Explain the scientific background and rationale for the investigation being reported | 5 |
| Objectives | | 3 | State specific objectives, including any prespecified hypotheses | 5 |
| Methods | | | | |
| Study design | | 4 | Present key elements of study design early in the paper | 5-6 |
| Setting | | 5 | Describe the setting, locations, and relevant dates, including periods of recruitment, exposure, follow-up, and data collection | 5-6 |
| Participants | | 6 | (*a*) Give the eligibility criteria, and the sources and methods of selection of participants. Describe methods of follow-up | - |
|  |  |  | (*b*) For matched studies, give matching criteria and number of exposed and unexposed | - |
| Variables | | 7 | Clearly define all outcomes, exposures, predictors, potential confounders, and effect modifiers. Give diagnostic criteria, if applicable | 6 |
| Data sources/ measurement | | 8 | For each variable of interest, give sources of data and details of methods of assessment (measurement). Describe comparability of assessment methods if there is more than one group | - |
| Bias | | 9 | Describe any efforts to address potential sources of bias | - |
| Study size | | 10 | Explain how the study size was arrived at | 6 |
| Quantitative variables | | 11 | Explain how quantitative variables were handled in the analyses. If applicable, describe which groupings were chosen and why | 6-7 |
| Statistical methods | | 12 | (*a*) Describe all statistical methods, including those used to control for confounding | 7 |
|  |  |  | (*b*) Describe any methods used to examine subgroups and interactions | 7 |
|  |  |  | (*c*) Explain how missing data were addressed | 6 |
|  |  |  | (*d*) If applicable, explain how loss to follow-up was addressed | - |
|  |  |  | (*e*) Describe any sensitivity analyses | 7 |
| Results | | | |  |
| Participants | | 13 | (a) Report numbers of individuals at each stage of study—eg numbers potentially eligible, examined for eligibility, confirmed eligible, included in the study, completing follow-up, and analyzed | 7-8 |
|  |  |  | (b) Give reasons for non-participation at each stage | - |
|  |  |  | (c) Consider use of a flow diagram | - |
| Descriptive data | | 14 | (a) Give characteristics of study participants (eg demographic, clinical, social) and information on exposures and potential confounders | 7-8 |
|  |  |  | (b) Indicate number of participants with missing data for each variable of interest | tables |
|  |  |  | (c) Summarise follow-up time (eg, average and total amount) | - |
| Outcome data | | 15 | Report numbers of outcome events or summary measures over time | 9 |
| Main results | 16 | (*a*) Give unadjusted estimates and, if applicable, confounder-adjusted estimates and their precision (eg, 95% confidence interval). Make clear which confounders were adjusted for and why they were included | | - |
|  |  | (*b*) Report category boundaries when continuous variables were categorized | | - |
|  |  | (*c*) If relevant, consider translating estimates of relative risk into absolute risk for a meaningful time period | | - |
| Other analyses | 17 | Report other analyses done—eg analyses of subgroups and interactions, and sensitivity analyses | | - |
| Discussion | | | | |
| Key results | 18 | Summarise key results with reference to study objectives | | 10 |
| Limitations | 19 | Discuss limitations of the study, taking into account sources of potential bias or imprecision. Discuss both direction and magnitude of any potential bias | | 13 |
| Interpretation | 20 | Give a cautious overall interpretation of results considering objectives, limitations, multiplicity of analyses, results from similar studies, and other relevant evidence | | 10-13 |
| Generalisability | 21 | Discuss the generalisability (external validity) of the study results | | 10-13 |
| Other information | | | | |
| Funding | 22 | Give the source of funding and the role of the funders for the present study and, if applicable, for the original study on which the present article is based | | 14 |

Note: An Explanation and Elaboration article discusses each checklist item and gives methodological background and published examples of transparent reporting. Information on the STROBE Initiative is available at http://www.strobe-statement.org.
